# A Wireless Rowing Measurement System for Improving the Rowing Performance of Athletes

**DOI:** 10.3390/s23031060

**Published:** 2023-01-17

**Authors:** Richard Hohmuth, Daniel Schwensow, Hagen Malberg, Martin Schmidt

**Affiliations:** Institute of Biomedical Engineering, TU Dresden, 01307 Dresden, Germany

**Keywords:** rowing, feedback training, movement analysis, surface EMG

## Abstract

The rowing technique is a key factor in the overall rowing performance. Nowadays the athletes’ performance is so advanced that even small differences in technique can have an impact on sport competitions. To further improve the athletes’ performance, individualized rowing is necessary. This can be achieved by intelligent measurement technology that provides direct feedback. To address this issue, we developed a novel wireless rowing measurement system (WiRMS) that acquires rowing movement and measures muscle activity using electromyography (EMG). Our measurement system is able to measure several parameters simultaneously: the rowing forces, the pressure distribution on the scull, the oar angles, the seat displacement and the boat acceleration. WiRMS was evaluated in a proof-of-concept study with seven experienced athletes performing a training on water. Evaluation results showed that WiRMS is able to assess the rower’s performance by recording the rower’s movement and force applied to the scull. We found significant correlations (*p* < 0.001) between stroke rate and drive-to-recovery ratio. By incorporating EMG data, a precise temporal assignment of the activated muscles and their contribution to the rowing motion was possible. Furthermore, we were able to show that the rower applies the force to the scull mainly with the index and middle fingers.

## 1. Introduction

Rowing is a technically demanding sport that requires a high level of endurance strength and motor coordination. To support the athletes, special measurement techniques were developed to verify and improve the movement of the athletes during training [1]. Errors in rowing technique can lead to an inefficient movement and can causes injuries [2,3]. Analysis of the rowing motion and feedback to the rower might prevent these injuries. New sensors and analysis methods allow measurement technologies to be further improved and provide the athlete with feedback in real-time [4,5]. The effectiveness of the training and ultimately the athletes’ performance can thus be further increased [6]. For this purpose, this study developed a novel measurement system that integrates multiple sensors to measure the athletes’ force and motion and sends it in real-time to a computer in the boat. The aim was to develop an easy-to-use modular measuring system that can be used on any rowing boat on water. Thus the sensors are fully integrated within the scull for measuring the force and the oar movement. Furthermore, the force exerted on the scull is measured with pressure sensors to obtain information about the rowers’ grip pattern on the handle. Another sensor unit is attached to the seat and the boat to measure its position and velocity. To validate the measurement technique, a proof-of-concept study was performed with seven experienced rowers. The recorded values of the sensors were compared with the state-of-the-art. Multimodal measurement systems are expected to be able to assess the rower’s performance better than state-of-the-art systems, so that feedback from such systems to the athlete will lead to performance optimization.

### 1.1. Rowing Technique and Challenges for Measurement

Rowing performance depends on technique as well as accuracy of execution. Rowing is a physically demanding sport in which the movements must be precisely coordinated [6]. During a rowing cycle, 80% of the body’s muscles are affected [3].

The rowing movement can be divided into four phases: (a) the catch defines, the moment when the oar blades enter the water. The athletes legs are in a flexed position with the upper body bent forward and the arms are extended; (b) during the drive phase the athlete generates propulsion. The rower pushes at the footrest, extends his legs and pulls the arms towards his chest; (c) when releasing, the legs are fully extended. The oar blades are lifted out of the water and the rower is furthest away from the catch position and (d) the recovery, where the athlete returns to the catch position with the oar blades are out of the water [7].

Rowing measurement systems allow the measurement of the movement and the analysis of the rowing technique. Studies used the measurement data, among other things, to analyze common errors in rowing technique. According to [5,7], the following examples can be listed in this regard:

1. Consistent rowing stroke length and pattern
2. Blade depth
3. Correct catch and finish
4. Relaxed recovery
5. Powerful, smooth movement overall

During the stroke, the boat’s acceleration has a characteristic curve that results from the execution of the rowing technique [8]. Errors in the individual rowing technique change the acceleration of the boat. In addition to the boat acceleration, the course of the scull force curve is also important [8,9]. There are various known rowing styles, such as Rosenberg-, DDR-, Adam- or Ivanov-style, which have different force curves [6]. To assess the rowing technique, it is necessary to adjust the movement to the respective rowing style. Rowers try to keep the boat speed constant during the rowing motion to minimize negative side effects. However, the boat speed during the rowing motion has a characteristic pattern in which the maximum boat speed is reached only after the recovery phase, because in this phase the mass of the rower moves in the opposite direction and the boat accelerates [8,9]. Deviations from the ideal behavior indicate errors in the rowing technique and lead to inefficient movement of the boat. Another parameter examined is the drive-to-recovery ratio. This parameter describes the ratio between the duration of drive and recovery phase. The theoretically optimal drive-to-recovery ratio is 1:2. The drive-to-recovery ratio decreases for high stroke rates. The recovery is important for the transfer of momentum from the rower to the boat. If this phase is too short, the effectiveness of rowing decreases [9]. Previous investigations of the maximum acceleration and deceleration showed that the boat decelerates more during the catch at higher stroke rates and accelerates less during the drive phase. Since the boat has a higher speed at higher stroke rates, the dipping of the oar blades during the catch has a significantly greater effect on the boat’s speed. Likewise, at higher boat speeds, there are higher frictional forces that have to be overcome in order to accelerate the boat [8].

The analysis of the oar angles gives a direct insight into the movement of the rower. Oar angles reflect the movement of the scull in the oarlock. This allows the detection of the rowing phases, as well as the trajectory of the scull [7] and also the investigation of typical errors in rowing technique, such as skying [5]. Different metrics can be examined to analyze the rowing movement. The correlation between stroke length and stroke rate provides information about the effectiveness of the movement [7]. Soper et al. [9] described a decreasing stroke length at higher stroke rates. This means that the full potential cannot be used during a stroke and the work done by the rowing stroke does not correspond to the theoretical maximum energy transmission.

The challenge for a measurement system is to capture the complex movement of the athlete as well as the movement of the boat. Therefore, the movement of the sculls in three-dimensional space, the resulting forces and the movement of the athlete within the boat need to be investigated. External influences such as currents and weather also have an effect on the reaction of the boat. Due to changing water levels on a river and the associated changes in the waterway, an evaluation of the measurement system for rowing is not useful by a single measurement. The measurement system has to cover a wide range of different environmental conditions.

### 1.2. State-Of-The-Art

Current measurement systems are distinguished regarding their intended use. On the one hand, there are systems that are intended for use in sports and for evaluation by the coach. These systems are characterized by an easy installation in the boat. The data is recorded by a computer unit and presented to the coach. Data processing often takes place in the system, which facilitates the trainer’s evaluation. Most common systems in rowing training is the instrumented oarlock, e.g., *Nielsen-Kellermann Co., Boothwyn, PA, USA, EmPower Oarlock*. These systems are attached to the boat as the oarlock, the connection between the scull and the boat. Instrumented oarlocks are able to measure oar angle and force with contact based methods, as well as all derived parameter like stroke length and power. Other available systems are mounted directly on the scull like *BioRow Ltd., Slough, UK, 4DHandle* or *WEBA Sport und Med Artikel GmbH, Vienna, Austria, Oar Power Meter* which are capable of measuring force using load cells and oar angle using IMUs. Through his experience, the coach decides how the athlete can improve the rowing technique. On the other hand, there are systems for scientific evaluation by sports physiologists. With these systems, the raw data of the movement and force application is recorded, like the systems described by Tessendorf et al. [7]. The scientists process the recorded data in a way that specific aspects of movement patterns become visible. The measurement setup is often complex, which means that assembly takes more time and allows rowers less access to such training equipment.

## 2. Materials and Methods

### 2.1. The Wireless Rowing Measurement System

**Figure 1:**
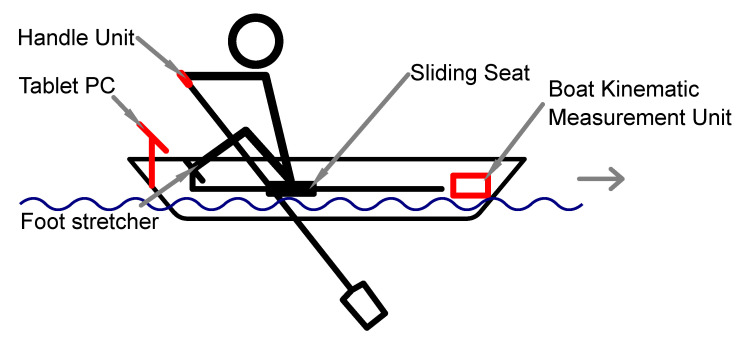
Structure of the developed wireless rowing measurement system (WiRMS) with the handle unit, the boat kinematic measurement unit and the tablet PC. Units were highlighted in red.

The wireless rowing measuring system (WiRMS) we developed and present in this paper consists of three units, see Figure 1. Each of the two sculls has a handle unit integrated. The boat kinematic measuring unit mounted behind the rower on the rowing boat, while the tablet computer is in the front of the rower on the footrest slides. Two measuring devices are wirelessly connected to the tablet PC via Bluetooth Low Energy (BLE). All three units are battery-powered, hence the entire system is cable free.

#### 2.1.1. Handle Unit

**Figure 2:**
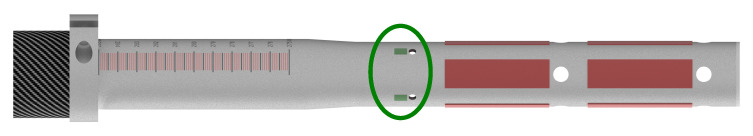
CAD rendering of handle unit with the surface pressure sensors (red) and strain gauges (green) mounted on a Concept 2 scull.

The developed handle unit is able to measure the spatial angles (pitch, yaw and roll), three axis acceleration and applied forces in two axes of the oars, see Figure 2. Furthermore, a two-by-four pressure sensor grid array is integrated on the handle to measure the pressure applied by the rower’s hand.

A custom build signal acquisition module was developed for the data collection. The module is based on an *Espressif System, Shanghai, PRC, ESP32* microcontroller with a *Bosch Sensortec GmbH, Reutlingen, Germany, BNO055* IMU and a precision ADC from *Texas Instruments, Dallas, TX, USA*. The forces were measured biaxially with strain gauges connected in a full Wheatstone bridge. The grip pressure is measured with a matrix of eight surface pressure sensors, based on force sensing resistors *Interlink Electronics Inc., Irvine, CA, USA, FSR UX408*.

#### 2.1.2. Boat Kinematic Measurement Unit

The developed boat kinematic measuring unit is able to capture the boats acceleration (three axis), the spatial angles and the position of the sliding seat relative to the measuring unit. It is also equipped with a GPS sensor. The measurement hardware is based on an *Espressif System ESP32* microcontroller with a *Bosch Sensortec BNO055* IMU as accelerometer. The sliding seat displacement is measured with a *STMicroelectronics N.V, Plan-les-Ouates, Switzerland, VL531L1X* time-of-flight sensor, and the GPS position is measured with a *CDtop Technology, Kaohsiung City, Taiwan, PA1010D* with 10 Hz sample rate. The boat kinematic measurement unit is attached to the boat hull with a suction cup. Further data processing ensures that there is no need to calibrate the system afterwards and the instrument is ready for use within a few minutes.

### 2.2. Electromyography—Muscle Activity

**Figure 3:**
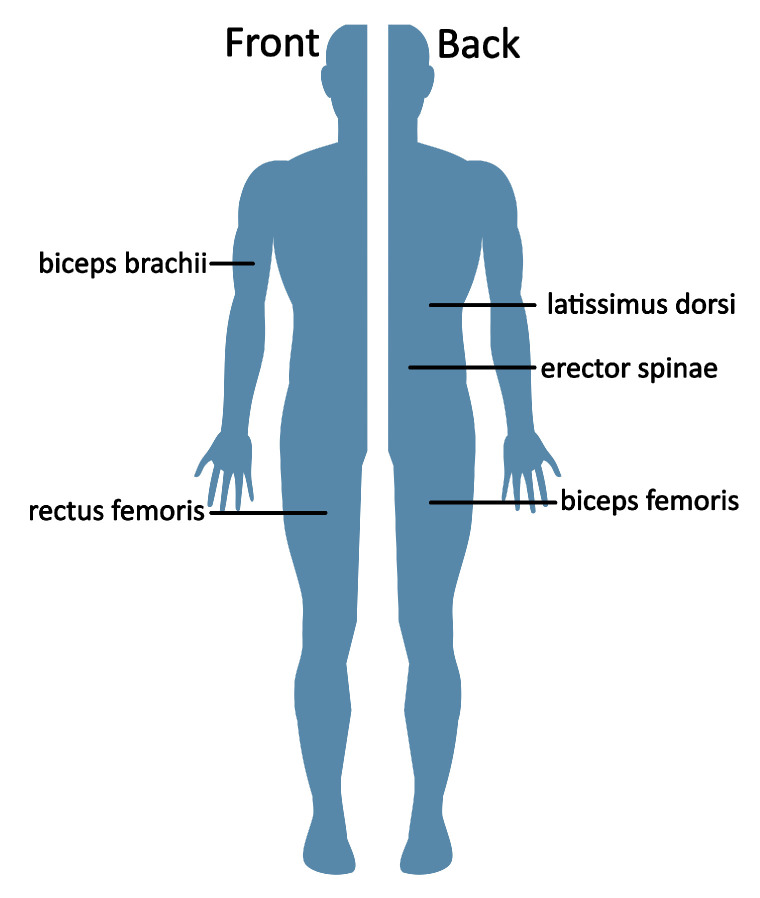
Muscles recorded with EMG sensors.

To achieve additional physiological effects, the extension with EMG sensors is foreseen [10,11]. For recording *Menios GmbH, Ratingen, Germany, MiniWave Plus waterproof* wireless EMG sensors with a sample frequency of 2 kHz were used. For data acquisition the data was stored on the EMG sensor and transferred to the tablet PC after the measurement. The signal processing is described by Schwensow et al. [11]. For this study we examined the following muscles: biceps brachii dexter, sinister; latissimus dorsi dexter, sinister; erector spinae dexter, sinister; rectus femoris dexter and biceps femoris dexter, see Figure 3. Two Ag-Ag/Cl surface electrodes (*Ambu GmbH, Bad Nauheim, Germany, BlueSensor N*, 2.2 cm in diameter) per sensor were placed according to the SENIAM recommendations (Surface Electromyography for the Non-Invasive Assessment of Muscles) [12].

### 2.3. Tablet PC

A *Microsoft Corporation, Redmond, WA, USA, Surface* tablet computer is used for data collection and to provide visual and acoustic feedback of the target stroke rate to the athlete. A custom build signal processing and visualization software based on *Unity* (*Unity Technologies, San Francisco, CA, USA*) was developed. The software establishes a connection between the sensor units and the tablet PC. The sensor data is received and stored in real-time on the PCs storage. The connection is made via BLE. This ensures a dependable and energy efficient connection between the sensor units and the tablet PC. Because the BLE standard does not require pairing between devices, the connection can be established quickly. For data transmission via BLE, a custom BLE service and BLE characteristic was developed.

### 2.4. Comparison to State-Of-The-Art Technology

In this section, we compare WiRMS features with systems already available on the market. While state-of-the-art systems measure scull forces in horizontal axis only [4,13], WiRMS can additionally measure vertical forces. Thus, WiRMS offers the possibility to analyze the lifting process, which is of particular interest in the catch and release phase. In addition, the grip pattern is measured, which for the first time provides information about the distribution of the pressure exerted by the athlete’s hands on the scull grip. The build-in IMUs allow to measure the oar angles in three axes. Sensors in the handle unit are fully integrated in the handle of a rowing scull, see Figure 2. All sensor units are completely wireless, and the seat displacement is measured contactless. To the authors’ knowledge, the measurement system developed is the first of its kind that allows data from an external EMG system to be synchronized with an on-board rowing measurement system. For this purpose, the time stamps of the individual systems as well as the data of the acceleration sensors installed in the measuring system and also in each EMG sensor are used. The systems were initially synchronized with each other and the maximum time deviation is one sample. A comparison between WiRMS and the state-of-the-art is shown in Table 1.

### 2.5. Rowing Parameters

WiRMS is able to record the movement and the force of the rower. Variables used to calculate the rower’s movement are the oar angles, grip forces, seat displacement, and boat acceleration and velocity, see Table 2. Simultaneous acquisition of different parameters enables both precise but also robust redundant acquisition of the rowing motion.

#### 2.5.1. Oar Angles

From the spatial angles of the oar, the stroke length, the immersion depth of the oar blades in the water and the rotation of the oar blades can be calculated. For the calculation of the oar angles, the IMU data recorded by the handle unit were used. The rotation around the z-axis is defined as the horizontal rotation and specifies the stroke length, as the angle between catch and release. The rotation around the x-axis defines the immersion depth of the blade and the y-axis defines the rotation of the scull. The above-mentioned measurements allow the investigation of common errors of rowing technique, which can lead to reduced effectiveness and thus to slower pace [6].

#### 2.5.2. Handle Force

The applied force is measured in horizontal and vertical axis. The pressure sensors within the handle unit enable measurement of the force exerted by the athletes’ hands on the scull. To detect the beginning of a stroke a force threshold of the rising edge was used. Between the strokes the data points were resampled to 1000 samples per stroke, after that, the average was calculated for each point in time of a rowing stroke.

#### 2.5.3. Seat Displacement

The seat displacement describes the position of the rower on the sliding seat. The used time-of-flight laser sensor measures the distance between the rower and the boat kinematic mesurement unit. The sensor is located in the boat kinematic measurement unit and directs a laser beam onto the seat of the rower. In this way, the distance between the seat and the boat kinematic measurement unit can be measured. The oar angles and the seat displacement are the key metrics to describe the rowers movement.

#### 2.5.4. Boat Acceleration and Velocity

The acceleration and speed of the boat is measured by the boat kinematic measurement unit. During the rowing movement, a characteristic acceleration pattern occurs (Section 1.1), which is measured by the acceleration sensor of the IMU. To assess the influence of currents and weather, the speed of the boat is measured with a GPS sensor.

#### 2.5.5. Muscle Activity

The muscle activity is derived from the electromyogram (EMG). By recording several muscles during rowing, the time at which the measured muscle fibre is activated can be determined. The respective muscle thus contributes to the total effective force that propels the boat. The amplitude of the EMG signal is proportional to the applied force [14]. However, the estimation of force by the EMG signal is still a research topic and not part of this study. Our results refer only to the activation pattern during the rowing stroke, but indicate the general feasibility of further analyses. For the evaluation of the muscle activity, the raw EMG signal was rectified and normalized to the maximum amplitude during the training course. Afterwards a moving root-mean-square (RMS) filter (window size = 100 samples, shift = 1) was applied.

### 2.6. Study Design

In order to evaluate WiRMS, seven experienced rowers volunteered for a test run on the Elbe River (Germany) with boats from the rowing club “Laubegaster Ruderverein Dresden e.V.”. The training course was coordinated with the trainer and is similar to the usual procedure in the club. The rowers completed a five-stage training course with different stroke rates and durations, see Table 3. Between the stages, the rowers had an individual resting period. The boat was equipped with the measurement sculls and the boat kinematic measuring device. All measurement systems were connected to the tablet PC, which was installed on the boat. The EMG sensors were placed on the rower’s body according to Section 2.2. The training started with a warm up period to get familiar with the modified scull. After that, the first stage of training began and ended with stage five (see Table 3). During the whole training session, the developed measuring system recorded all data on the tablet PC in real-time.

## 3. Results

**Figure 4:**
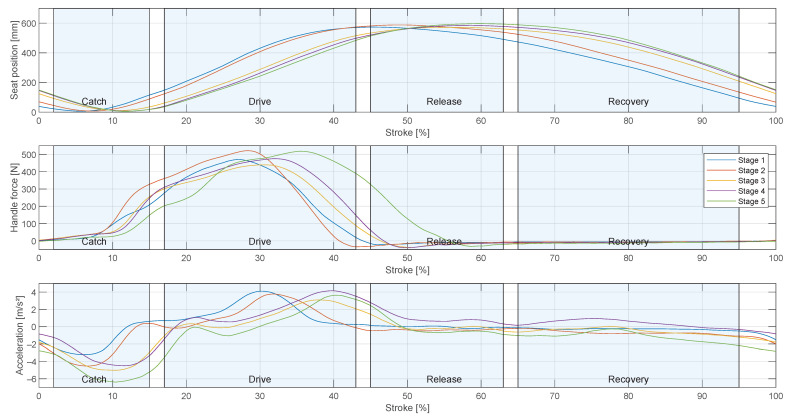
Seat movement, horizontal oar angle and force over average rowing stroke curve for different stages during the training. Blue areas mark rowing phases.

Figure 4 shows the average movement exemplary for participant 3 on the sliding seat, as well as the applied force to the scull and the boat acceleration during each stage of the test protocol (Table 3). With the presented data, a basic assessment of the athlete’s movement and the resulting effect on the boat can be made. For the evaluation of rowing strokes, the first four strokes were excluded, as the rower had to overcome the inertia of the boat. To detect the beginning of a stroke, a force threshold of the rising edge was used. For the classification of the rowing phases the seat position was used as the distance of the sliding seat to the most forward position during a stroke. The rowing stroke starts with the catch, the rower is in the most forward position. The drive phase is initiated with rapid increasing forces measured at the handle. Maximum force is reached between 27% and 35% of the stroke, depending on the stroke rate. The rower reaches a maximum force between 450 N and 510 N in average. At the end of the drive phase, the force decreases till the release between 40% to 57% of the stroke, depending on the stroke rate. The rower is now in backward position and the oars are in the most forward position where they leave the water in the release position. Because of the flexion of the oars, the force is slightly negative, as the blade is outside of the water this negative force does not decelerate the boat. During the recovery phase, the sculls remain outside the water and the rower returns to the catch position. The rower transfers his momentum from the drive phase to the boat, which can lead to a positive boat acceleration (stage 4) or prevents the boat from decelerating. However, other environmental factors also have an effect on boat acceleration, see Section 4. Meanwhile, no force is acting on the scull.

**Figure 5:**
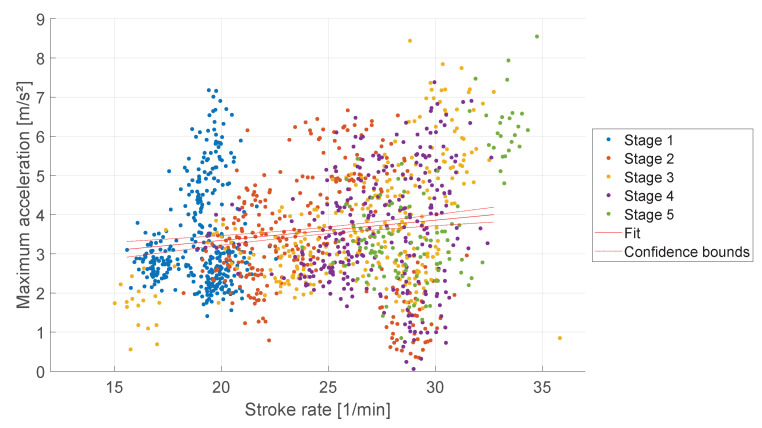
Maximum acceleration over stroke rate during five stage rowing training. A linear regression fit, and 95% confidence interval is marked in red.

**Figure 6:**
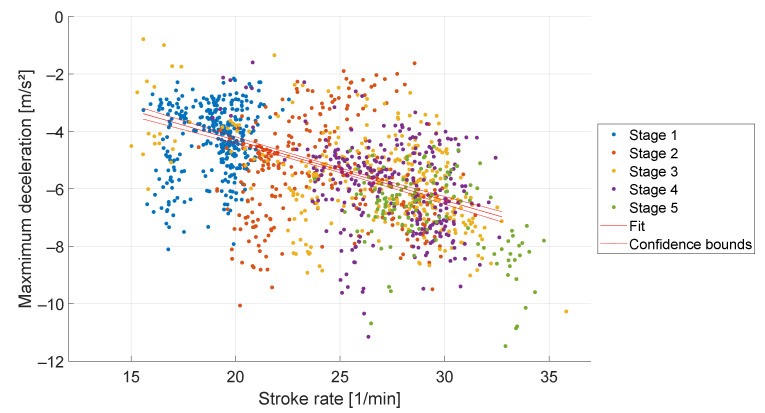
Minimum acceleration over stroke rate during five stage rowing training. A linear regression fit, and 95% confidence interval is marked in red.

The boat acceleration is a characteristic parameter for assessing rudder performance, see Section 1.1. Figure 4 shows the average boat acceleration during the five load stages. The rowing stroke starts with a deceleration of the boat because the oar blade causes friction in the water, followed by an acceleration when the rower applies the force to the oar blade. At higher stroke rates, the peak acceleration during the rowing stroke is later, see Figure 4. The average peak of the first load stage occurs at 30% of the rowing stroke with 4.3 m/s^2^ and in the fifth load stage at 41% with 3.8 m/s^2^. Figure 5 shows a low linear correlation between the stroke rate and maximum acceleration with a slope of 0.05 m· min/s^2^ (R^2^ = 0.02, *p* < 0.001). The deceleration begins earlier during higher stroke rates and decelerates more compared to lower stroke rates, see Figure 6. A linear correlation was detected with a slope of −0.21 m·min/s^2^ (R^2^ = 0.29, *p* < 0.001).

**Figure 7:**
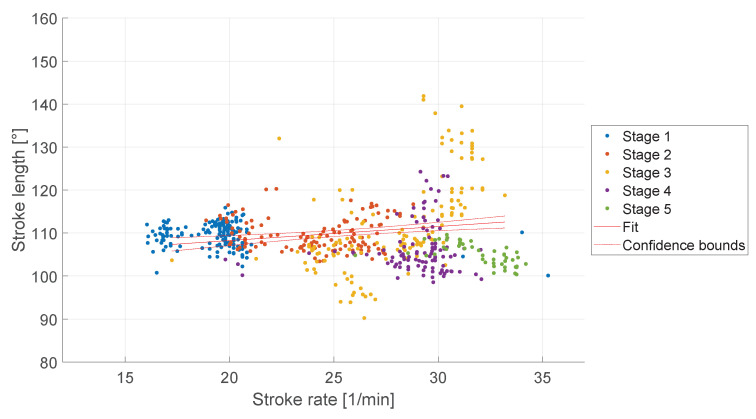
Stroke length and stroke rate of the rower during five load stages.

Stroke length, or the distance covered by the oar during each stroke, can have an effect on rowing performance. As described in Section 1.1 the stroke length can be influenced by the stroke rate. Figure 7 shows the stroke length compared to the stroke rate of the rowers during the five load stages. For the evaluation of the stroke length, the data of only three participants were suitable, due to a configuration error of the IMU sensors inside the sculls. Therefore, the data of the first four participants had to be excluded. During stage three, the standard deviation for the stroke rate (2.85 spm) and the stroke length (10.76°) were higher than during the other stages, see Table 4. A linear regression showed a dependence of the stroke rate on the stroke length (R^2^ = 0.038, *p* < 0.001). However, due to the low R^2^-value, the linear model could explain only 3.8% of the variability in the stroke length.

**Figure 8:**
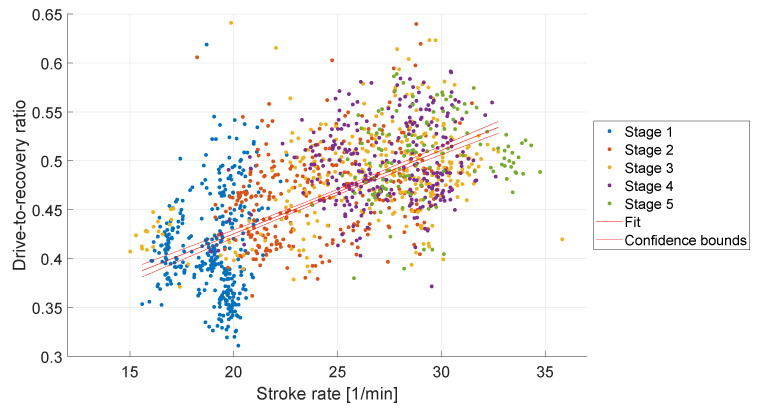
Drive-to-recovery ratio compared to the stroke rate during five stage rowing training. A linear regression fit and 95% confidence interval is marked in red.

The drive-to-recovery ratio is a number used to evaluate rowing performance and is influenced by the stroke rate, as described in Section 1.1. Figure 8 shows the drive-to-recovery ratio compared to the stroke rate that the rowers reached during the training. The drive-to-recovery ratio increases with increasing stroke rates. In this study, drive-to-recovery ratios of 0.31 to 0.65 were determined. A proportional increase in the drive-to-recovery ratio with the stroke rate was observed with a slope of 0.01 min/1 (R^2^ = 0.37, *p* < 0.001).

**Figure 9:**
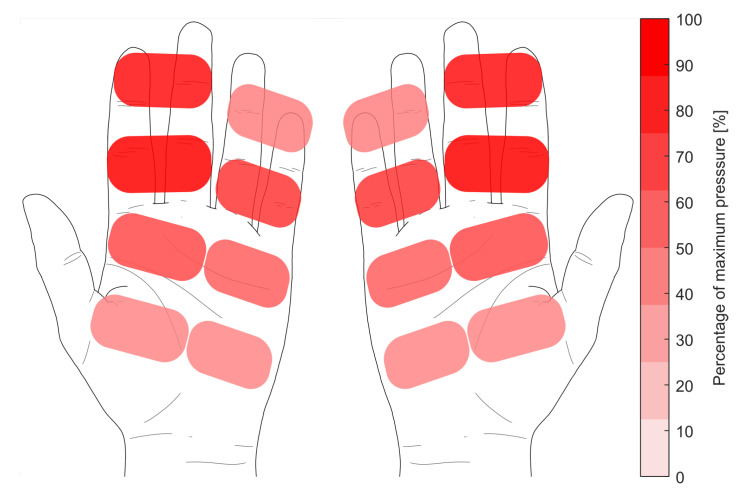
Pressure map of the rowers hands during the drive phase.

The force is applied to the scull by the athlete via the grip surface. This is detected by pressure sensors on the surface of the grip. Figure 9 shows the pressure distribution of the hand on the scull during the drive over all participants. The pressure sensors embedded in the handle record the force that the rower applies to the scull. It can be seen, that the force is mainly applied with the index and middle finger (average of 86.45% of the maximum applied pressure at maximum force), while the other sensors reaches only 31.68% in average during the peak force application. The pressure distribution is calculated as the percentage of the highest pressure measured during a randomly selected drive phase.

**Figure 10:**
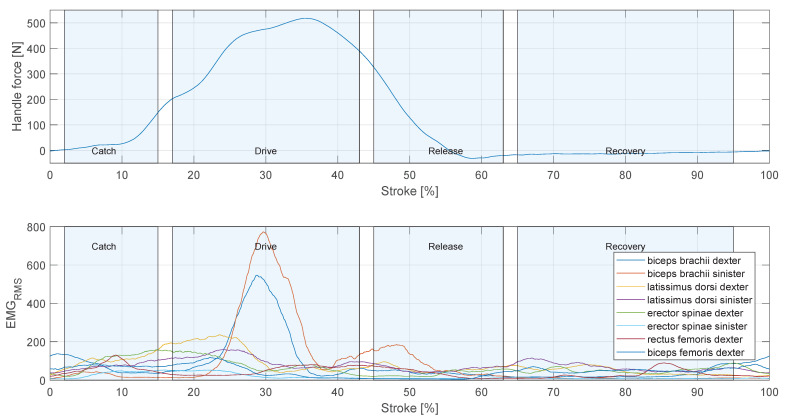
Activation pattern of monitored muscles during an average rowing stroke. $EMG_{RMS}$: root-mean-squared EMG signal.

Rowing is a complex movement involving multiple muscle groups (Section 1.1). EMG was used to reconstruct the temporal structure of muscle activation, see Section 2.5.5. Figure 10 shows the muscle activation pattern of participant 3 during the averaged rowing stroke in stage 5. It can be seen, that the muscle activity rises during the drive phase and remains low during the recovery phase. In the beginning of the stroke, there is still activity in the m. biceps femoris remaining from the end of the recovery phase. Followed by activity of the m. latissimus dorsi and m. erector spinae. At 30% of the stroke, during the drive phase, there are prominent peaks in the signal of the m. biceps brachii dexter and m. biceps brachii sinister. During the release phase is second peak in the activity of the m. biceps brachii sinister is evident.

## 4. Discussion

The WiRMS developed and presented in this paper is a prototype. For evaluation purposes, a proof-of-concept study was performed in which the results were compared with similar investigations from the state-of-the-art technology. The study was conducted with a seven participants on different dates over a period of four weeks. Therefore, the results considering only the rowing technique of the individual athletes and are dependent on the respective environmental conditions. A measurement with a reference system was not performed in this first proof-of-concept study, but will be considered in further investigations.

An important part of the analysis of the rowing movement is the detection of the stroke phase. In this study, the position of the sliding seat was used for the detection. For the detection of the catch phase, a threshold in the rising force edge was used.

Kleshnev [8] investigated the temporal structure of the boat acceleration. In his study, a proportional increase in boat acceleration and stroke rate was found, which is consistent with the results of our study, see Figure 5. The proportional decrease of the deceleration observed in this study, see Figure 6, was also documented by Kleshnev.

Soper et al. [9] describe the optimal ratio of drive and recovery as 0.33. In the studies they describe the drive-to-recovery ratios of 0.37 to 0.53 were found, whereby the drive-to-recovery ratio increases with increasing stroke rate. This can be shown in this evaluation, see Figure 8. We observed a mean drive-to-recovery ratio of 0.46 in our study.

The rowing movement is initiated by the m. biceps femoris, this can be seen in Figure 10. The activity of m. biceps femoris starts at the end of the recovery phase as the rowers returns to the catch position and had to decelerate the sliding seat. Compared to Pollock et al. [15], the activity of m. biceps femoris at the beginning of the movement is as expected. Pollock et al. also describe a second peak in the activity of m. biceps femoris at 20% of the stroke, this can not be confirmed in our study. M. latissimus dorsi and m. erector spinae show a higher activity from the start of the rowing stroke till 30% of the stroke, this is in accordance with the observations of Pollock et al. As mentioned in Section 2.5.5, the force produced by the muscles is in correlation to the amplitude of the respective EMG signal. However, the EMG signal can not be compared among different muscles. Thus, the different amplitude in the activation of m. biceps brachii dexter and sinisiter does not result in different forces on the left and right scull.

In order to record values that are as realistic as possible, one of the considerations in developing the measuring system was that the rower should not notice the measuring devices. The handle equipped with the measurement technology does not differ from the original in appearance. Nevertheless, the participants reported after the training that rowing with the modified scull felt different because of the slightly additional weight. This can lead to deviations in the rowing movement. However, as there are no recordings of the rowers with a reference system, no statement can be made about the impairment in this study. Measurement of the performance improvement from feedback of WiRMS to the rower was not part of the proof-of-concept study, but will be investigated in a further larger study.

Our analyses showed that the developed measurement system provides data comparable to other state-of-the-art systems. With the rowing parameters described in Section 2.5, it could be shown that the developed measurement system is suitable for the performance assessment of rowers in our study. This approves the performance of the developed system as a basis for further investigations.

## 5. Conclusions

Within the scope of this work, a powerful wireless measuring system (WiRMS) for recording and evaluating the rowing movement was developed. The measuring system consists of two modified sculls with integrated sensors in the handle. The handle unit is able to measure the movement of the handle and thereby calculate the oar angles. It also measures the forces on the handle and the pressure distribution on the handle surface. Additionally, a boat kinematic measurement unit, which is attached to the rowing boat, and a tablet PC for recording and visualizing the measurement are integrated. The boat kinematic measurement unit measures the movement of the boat with acceleration and GPS sensor. In addition, the position of the rower on the sliding seat can be measured contactless. Furthermore, muscle activity can be measured by means of a simultaneously recorded EMG. To the authors’ knowledge, the developed system is the first of its kind to allow simultaneous measurement of external EMG data synchronized with an on-board rowing measurement system.

A proof-of-concept study was conducted to evaluate the measurement technique and the results were compared with similar studies from the state-of-the-art. It was shown that the measuring system is capable of recording the defined parameters to evaluate the rowing movement and thus the data is suitable to assess the rowing performance.

## 6. Outlook

The developed measurement system provides a basis for further investigations. A study with more subjects is needed to build a diverse database. Additionally, it is necessary to compare the accuracy of the developed WiRMS with a reference rowing measurement system. This recorded database will be used to create a model on which the athletic performance of the rowers can be measured and evaluated. For this purpose, appropriate quality criteria for the rowing technique have to be defined. The model would allow the athlete to receive automated feedback on how to improve his rowing technique. In this way, individual rowing performance can be improved and injuries to rowers can be prevented.

## Figures and Tables

**Table 1:** Comparison of different system parameters between the state-of-the-art and WiRMS.

Parameter	State-Of-The-Art ^1^	WiRMS ^1^
Oar Angle	3D	3D
Handle Force	1D	3D
Grip Pressure	No	8 areas per handle
Seat Displacement	Contact based	Contactless
Boat Acceleration	Yes	Yes
Boat Velocity	Yes	Yes
Muscle Activity	No	Yes

^1^ 1…3D—one…three-dimensional, WiRMS—wireless rowing measurement system.

**Table 2:** Rowing Parameters recorded by WiRMS.

Parameter	Sensor	Unit
Oar Angle	IMU	Degree [°]
Handle Force	Strain gauge	Newton [N]
Grip Pressure	Force sensing resistor	Pascal [Pa]
Seat Displacement	Laser time-of-flight sensor	Meter [m]
Boat Acceleration	IMU ^1^	Meter per second squared [m/s^2^]
Boat Velocity	GPS ^2^ sensor	Meter per second [m/s]
Muscle Activity	EMG sensor	Microvolt [µV]

^1^ IMU—inertial measurement unit, ^2^ GPS—gloabl positioning system.

**Table 3:** Load stages for five-stage rowing training.

Stage	Stroke Rate [Strokes per Minute]	Duration [s]
1	20	180
2	22	120
3	24	120
4	26	120
5	28	60

**Table 4:** Average and standard deviation of stroke length and stroke rate of all rowers during five load stages.

Stage	Stroke Rate [spm]	Stroke Length [°]
1	19.35 ± 2.47	109.55 ± 2.84
2	24.12 ± 2.55	109.68 ± 3.71
3	27.64 ± 2.85	111.87 ± 10.76
4	28.97 ± 2.01	106.38 ± 5.86
5	31.84 ± 1.7	105.18 ± 2.54

## Data Availability

Not applicable.

## Abbreviations

The following abbreviations are used in this manuscript:

WiRMS	Wireless rowing measurement system
EMG	Electromyogram
BLE	Bluetooth Low Energy
IMU	Inertial measurement unit
GPS	Global positioning system
